# Simulation of Light Distribution in Gamma Irradiated UHMWPE Using Monte Carlo Model for Light (MCML) Transport in Turbid Media: Analysis for Industrial Scale Biomaterial Modifications

**DOI:** 10.3390/polym13183039

**Published:** 2021-09-09

**Authors:** Ali Rizwan, Muhammad Saleem, Suhail H. Serbaya, Hemaid Alsulami, Aqsa Ghazal, Malik Sajjad Mehmood

**Affiliations:** 1 Department of Industrial Engineering, Faculty of Engineering, King Abdulaziz University, Jeddah 21589, Saudi Arabia; arkhan71@kau.edu.sa (A.R.); sserbaya@kau.edu.sa (S.H.S.); healsulami@kau.edu.sa (H.A.); 2 Department of Industrial Engineering, Faculty of Engineering-Rabigh, King Abdulaziz University, Jeddah 21589, Saudi Arabia; msaleim1@kau.edu.sa; 3 Department of Basic Sciences, University of Engineering and Technology, Taxila 47050, Pakistan; aqsaghazal06@gmail.com

**Keywords:** UHMWPE, chain mobility, photon distribution, Kubleka–Munk model, Monte Carlo simulation, gamma irradiations

## Abstract

(1) Background: This study investigated the miscibility of carbon-based fillers within industrial scale polymers for the preparation of superior quality polymer composites. It focuses on finding the light distribution in gamma irradiated ultra-high molecular weight polyethylene (UHMWPE). (2) Methods: The Kubleka–Munk model (KMM) was used to extract the optical properties, i.e., absorption coefficients (μ_a_) and scattering coefficients (μ_s_). Samples amounting to 30 kGy and 100 kGy of irradiated (in the open air) UHMWPE from 630 nm to 800 nm were used for this purpose. Moreover, theoretical validation of experimental results was performed while using extracted optical properties as inputs for the Monte Carlo model of light transport (MCML) code. (3) Conclusions: The investigations revealed that there was a significant decrease in absorption and scattering coefficient (μ_a_ & μ_s_) values with irradiation, and 30 kGy irradiated samples suffered more compared to 100 kGy irradiated samples. Furthermore, the simulation of light transport for 800 nm showed an increase in penetration depth for UHMWPE after gamma irradiation. The decrease in dimensionless transport albedo  μs(μa+μs) from 0.95 to 0.93 was considered responsible for this increase in photon absorption per unit area with irradiation. The report results are of particular importance when considering the light radiation (from 600 nm to 899 nm) for polyethylene modification and/or stabilization via enhancing the polyethylene chain mobility.

## 1. Introduction

In recent decades, industrial applications of polymers and polymer composites have seen an exponential rise in different areas of our life, including pharmaceutical packaging, consumer goods, medical devices, food and cosmetic packaging, etc. Nowadays, the medical devices used for implant replacement are mostly fabricated with ultra-high molecular weight polyethylene (UHMWPE) and/or its carbon-based composites [1,2,3]. UHMWPE is the material of choice for orthopedic industrial applications and medical devices that are made of this industrial scale polymeric biomaterial are usually treated with high energy radiation such as gamma, e-beams, or X-rays for sterilization [4,5,6,7,8,9,10,11]. However, treating UHMWPE with high energy radiation also generates free radicals that are responsible for its degradation, thus limiting the service life of the UHMWPE-based medical devices. To quench these free radicals, different methodologies including post-irradiation melting, post-irradiation annealing and the inclusion of biocompatible antioxidants are in practice. Although the aforementioned methodologies work well to some extent, serious constraints exist for each method. Therefore, continuous efforts are in progress to figure out the best alternative for sterilization and stabilizing UHMWPE. In this regard, the concept of treating UHMWPE with light [12,13] of suitable wavelength and/or energy seems to be appealing as it has the potential to address the harms associated with the aforementioned methods. Moreover, the non-ionizing nature of light also reduces the health risk for workers associated with the radiation sterilization industry of medical products. However, when using light to modify, sterilize and/or stabilize UHMWPE [13,14,15], an investigation of optical properties (i.e., absorption coefficient μa, scattering coefficients μs in pristine and irradiated UHMWPE [10,16] and light distribution in pristine and irradiated UHMWPE [17,18,19,20,21]) needs to be conducted.

High power lasers have been used in the recent past for surface modifications of UHMWPE, e.g., Fernández-Pradas et al. [14] used a pulsed laser (1027 nm, 450 fs @ 1 kHz) for surface modification of UHMWPE, and found that pulses of 6 μJ has the highest ablation efficacy as compared to low energy pulses. Riveiro et al. [13] investigated laser irradiated UHMWPE samples and tried to optimize the process parameters for various pulsed lasers, i.e., 1064 nm, 532 nm, and 355 nm, to maximize the surface properties. In another study, Hussain et al. [22] carried out laser texturing on UHMWPE surface while using 1024 nm pulsed laser at various temperatures. More recently, Ullsperger et al. [23] utilized a high power pulsed laser to enhance the mechanical properties of UHMWPE while fusing the UHMWPE particles with 500 fs laser pulses of 1030 nm. In this study, they were able to achieve better values of ultimate tensile strength by controlling the pulse energy and repetition rate. Although above mentioned studies are important for surface modifications/texturing of UHMWPE, the choice of laser wavelength has been done without considering the fundamentals of light-matter interaction, i.e., the optical properties and light distribution characteristics of UHMWPE.

A significant amount of work has been done to investigate the optical properties and distribution of light in soft materials and various tissues [24,25,26]; however, UHMWPE’s optical behavior has seldom been investigated. In research, Hamna et al. [20] highlighted the effects of radiation on spectroscopic properties, direct/indirect transition, and absorption behavior for e-beam irradiated UHMWPE. In another study, Asif et al. [27] concluded the anisotropic nature of UHMWPE in the UV-VIS region and confirmed the forwarded scattering nature of UHMWPE for UV-VIS light. Furthermore, the literature reveals a linear dependence between direct Urbach energy (E_u_) band gaps and absorbed dose [28]. More recently, Noor Us Saba et al. [29] studied the light distribution in UHMWPE for 300 nm and 800 nm and found that photons with low energy/higher wavelengths penetrate by a greater distance. The results reported in this study confirmed the fact that light of longer wavelength was suitable for UHMWPE bulk modification and/or treatment. Although the results reported by [29] were hopeful, unfortunately these were only for pristine UHMWPE.

The main objective of this study is to figure out the distribution of light so that it can be used to modfiy and/or stablize the irradiated UHMWPE. It is well established that radiation induces free radicals that are trapped within the UHMWPE matrix and can be eliminated via enhancing polyethylene long chain moblity and treating the irradiated components of UHMWPE with light of suitable wavelength and energy. However, knowledge about the distribution of light within the matrix of UHMWPE is required in order to achieve the aformentioned objetive. Therefore, this particular study is aimed at investigating the effect of radiation treatment on the measured values of diffuse reflectance, transmittance, and absorption of light from UHWMPE from 600 nm to 800 nm. The choice of light wavelengths is because of the proposed study theme where utilization of light is required for bulk treatment of UHMWPE. The optical properties, including the absorption coefficient (μa), scattering coefficients (μs), total attenuation coefficients (μt), and effective attenuation coefficients (μeff), were measured and then simulated to explore the effect of radiation on the light distribution.

## 2. Materials and Methods

### 2.1. Material and Sample Preparation

Medical grade UHMWPE powder (having molecular weight 3–6 million g/mol) purchased from Sigma Aldrich^®^ Saint Louis, MO, USA (Product Number: 434264 CAS Number: 9002-88-4 MDL: MFCD00084423 Formula: C_2_H_4_) was used in this study. Samples were molded in the form of thin sheets by using an automated hot press. UHMWPE powder pressing was done at 200 bars with a holding time of 12–15 min at 140 °C, 160 °C, and 190 °C, respectively. During the process of molding, the cool-down and ramp-up rate was set to 10 °C/min. After preparation, the thickness was measured and it was found that each sheet has a thickness equal to 500 ± 10 μm. Before measuring the thickness, each sheet surface was wiped off with acetone to remove the impurities.

### 2.2. Sample Irradiation

After the preparation of sheets, samples were divided into three groups. One was kept on the shelf as control and the other two sheets were sent for irradiation. Irradiation services were carried out (on payment) at Pakistan Radiation Services (PARS), Lahore while using the Co-60 source for 30 kGy and 100 kGy of gamma dose at a constant dose-rate of 1.02 kGy/h in open air at room temperature. After radiation treatment, the sheets were labeled P-0, P-30, and P-100. It is worth mentioning here that the dimensions of each sheet sent for irradiation were 10 cm × 10 cm × 500 μm, which was then cut to make three or four samples for testing. The sample codes mentioned above were used throughout the manuscript for explaining the results. The radiation facility used is used for providing sterilization services to food and medical devices and is constantly monitored by the Pakistan Nuclear Regulatory Agency (PNRA) for its reliability, safety, and environmental homogeneity.

### 2.3. Measurements of R_d_ & T_d_

A diffuse reflectance spectrometer (DRS) setup (UV/VIS/NIR spectrometer Lamda 950, integrated sphere 150 mm) purchased from PerkinElmer Life and Analytical Sciences (710 Bridgeport Avenue Shelton, CT 06484-4794, Waltham, MA, USA), equipped with an integrated sphere 150 mm with the spectral range from 250 nm to 1000 nm, was used for testing the samples. The reflectance and transmittance measurements were carried out at room temperature, i.e., 25 °C. Three or four readings were taken for each measurement and the average for each sample was then plotted as a function of wavelength and absorbed dose. The standard deviation for each measured data was negligible, i.e., ≤3%.

## 3. Results and Discussion

### 3.1. Effect of Radiation on Measurable

This research is aimed at investigating the light distribution in pristine and gamma-irradiated UHMWPE. To investigate this, diffuse reflectance and transmittance from 600 nm to 800 nm were measured, and the Kubleka–Munk model (KMM) was employed to extract the optical properties. The efficacy of the method for extracting the optical parameters, i.e., scattering coefficients (μ_s_) and absorption coefficient (μ_a_) in cm^−1^ was then validated theoretically with the Monte Carlo simulation. After validation, the obtained parameters were then used as inputs for obtaining the detailed picture of light distribution in pristine and irradiated UHMWPE.

**Figure 1:**
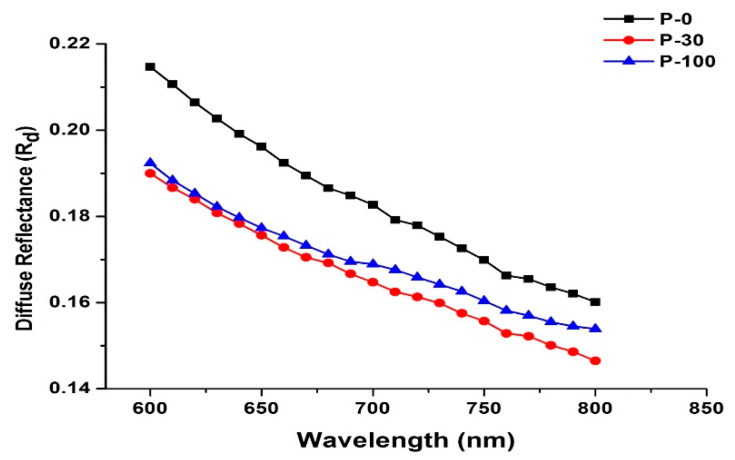
Experimental values of diffuse reflectance over the spectral range of wavelength from 600 nm to 800 nm.

Shown in Figure 1 are the variations of diffuse reflectance as a function of wavelengths for pristine, 30 kGy, and 100 kGy irradiated samples, respectively. The following trends are evident from the figure:

- Diffuse reflectance decreases as a function of incident wavelength for all samples, whether it is irradiated or un-irradiated.
- The amount of reflectance is higher in the visible range and lower in the near-infrared region.
- The amount of diffused reflected light is significantly reduced for 30 kGy and 100 kGy irradiated samples.

The factors responsible for reflectance of incident light from highly scattering materials like UHMWPE are:

- Surface roughness of the sample
- Refractive index mismatch
- Sample thickness

The diffusively reflected light is a combination of numerous refractions, reflections, and diffractions before being detected as diffuse reflectance. Furthermore, it also includes specular reflectance [29] in addition to the abovementioned contributions [30]. It is well established that irradiating UHMWPE in the open air results in an increase in polyethylene crystallinity [31,32,33,34]. Moreover, results reported in the literature [35] have shown that an increase in the crystallinity of the polymer composites leads to an increase in optical absorption, which is the main reason for the increase in absorption and lower value of diffuse reflectance for irradiated samples at each wavelength of interest during this study. The scattering of incident light from radiation-induced trapped free radicals within the UHMWPE matrix might be responsible for slightly higher values of R_d_ for the 100 kGy sample as the concentration of trapped free radicals is higher [36]. The higher values of R_d_ (as shown in Figure 1), larger penetration depth at longer wavelength, and larger concentration of free radicals for 100 kGy samples also support this argument [37,38,39].

**Figure 2:**
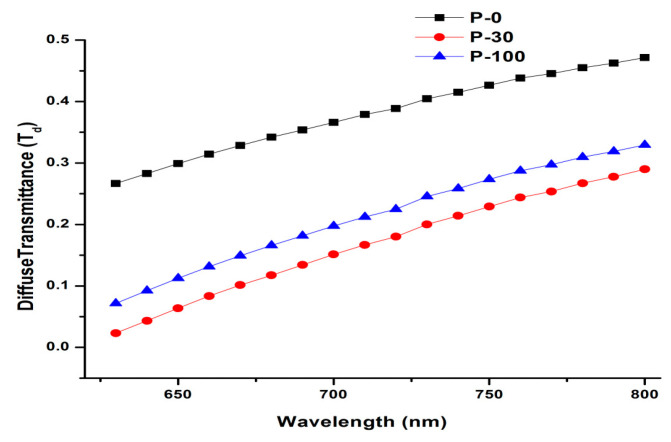
Variation in diffuse transmittance by increasing the wavelength from 600 nm to 800 nm with radiation dosage 0, 30 kGy, and 100 kGy.

The T_d_ trends differ from the R_d_ trends reported above in two respects: (1) T_d_ increases as a function of incident wavelength for all samples, whether irradiated or not; and (2) T_d_ values for 100 kGy irradiated sample is higher when compared with 30 kGy irradiated samples (see Figure 2). The measured values of diffuse transmittance depends on light photons that are scattered in the forward direction and the forward-directed scattering nature (in the optical window) of UHMWPE has been well documented in recent years [27]. Therefore, the increase in T_d_ as a function of incident wavelength is quite understandable; however, the behavior T_d_ for 100 kGy irradiated (highlighted above) is because of higher values of cross-linking networks for the 100 kGy irradiated samples [40]. It is well documented that the cross-linking networks within the UHMWPE matrix are responsible for aligning the randomly distributed crystalline lamellae in the amorphous slough of CH_2_ units [17,41,42,43,44]. Therefore, the 100 kGy irradiated sample is more oriented and transparent as compared to the 30 kGy one, which is observed experimentally (see Figure 2).

**Figure 3:**
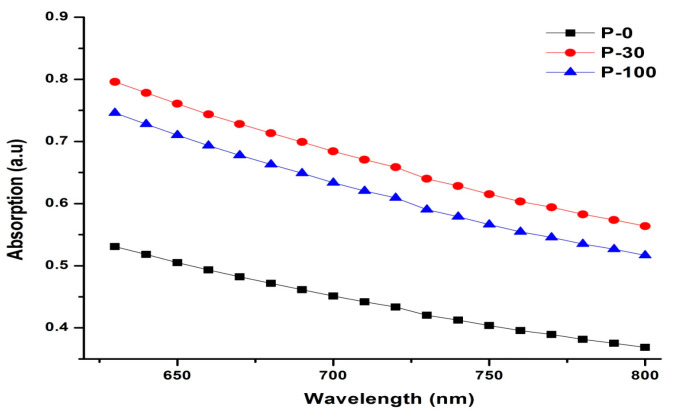
Experimentally measured values of absorption from UHMWPE sample over the spectral range of interest, i.e., 600 nm to 800 nm.

The trends for absorption obtained from the values of transmittance and reflectance are shown in Figure 3. The absorption values at lower wavelengths are higher as compared to higher wavelengths, and there is a significant increase in absorption values at each wavelength after irradiation. The increase in absorption is higher for the 30 kGy samples compared to the 100 kGy samples. This increase in absorption due to radiation-induced treatment is responsible for the reorganization of the chain by scission due to irradiation because of higher oxidative damage in the 30 kGy sample [28,31] is one of the major factors responsible for the observed higher values of absorption for the 30 kGy sample. Furthermore, the role of higher crosslinking yield in the 100 kGy irradiated sample (which is responsible for aligning and making the polymer matrix more oriented and transparent in a given wavelength range of interest) cannot be ruled out here.

### 3.2. Extraction of Optical Properties 

In the study, the DRS approach was implemented for the extraction of optical properties from measurements because of its simplicity and feasibility for the aforementioned purpose. DRS has the potential to quantify scattering and absorption properties related to highly scattering materials like UHMWPE [29]. To extract UHMWPE optical characteristics, i.e., absorption coefficient μ_a_ and μ_s_ for each sample used in this study, the two flux Kubleka–Munk model (KMM) is utilized, which utilizes the measured values of R_d_ and T_d_ and sample thickness. The following equations are used to figure out the optical characteristics of pristine and irradiated UHMWPE

(1) $$S=\frac{1}{\mathrm{yt}}[\frac{1-R_{d}\left(x-y\right)}{T_{d}}]$$

(2) µa=K2, µs=4S+µa31−g, K=Sx−1

(3) $$x=\frac{\left[1-T_{d}{}^{2}+R_{d}{}^{2}\right]}{2R_{d}}\;,\;\;y=\sqrt{x^{2}-1}$$

where

- K is the flux loss per absorption per unit length
- S is the scattering per unit length
- R_d_ is the measured value of diffuse reflectance
- T_d_ is the measured value of diffuse transmittance
- t is the sample thickness
- g is the anisotropy factor and its value is taken as 0.9 due to the forwarded directed scattering nature of UHMWPE [27,28]

### 3.3. Effect of Radiations on Extracted Optical Properties

The extracted optical properties for pristine, 30 kGy, and 100 kGy samples corresponding to each incident wavelength are tabulated in Table 1. The following trends of absorption coefficients (μ_a_) and scattering coefficients (μ_s_) are obvious as a function of the increase in incident wavelength and absorbed dose.

- For an un-irradiated sample, μ_a_ increases from 5.34 cm^−1^ to 6.09 cm^−1^, and μ_s_ increases from 120.06 cm^−1^ to 128.02 cm^−1^ over the wavelength of interest, i.e., 630 nm to 800 nm. The higher the incident wavelength, the longer the mean free path is before each interaction, so the abovementioned increase in μ_a_ and μ_s_ (as a function of wavelength) for the pristine sample is quite understandable.
- There is a significant decrease in the coefficient (μ_a_ & μ_s_) values from 630 nm to 700 nm for irradiated samples. Furthermore, it can be seen from the table that the decrease in coefficients for a 30 kGy irradiated sample is higher as compared to a 100 kGy irradiated one, which is due to greater oxidation damage [17,37,38,40] for the 30 kGy samples, as mentioned above.
- There is a slight increase in the values of μ_a_ on moving from 700 nm to 800 nm for irradiated samples, which is attributed to the dominant scattering and small absorption of polyethylene on near IR and IR absorption [27,30]. The decrease in per unit scattering length, i.e., μ_s_ from 700–800 nm and the significant increase in T_d_ for irradiated samples (see Table 1 and Figure 2) also supports this argument.

The decrease in absorption coefficient is dominant in the lower wavelength region for 30 kGy irradiated samples (see Table 1). Similarly, there is a notable decrease in scattering coefficients, which is evidence of the fact that irradiating the samples is responsible for decreasing the absorption and scattering coefficients of the samples. However, an increase in the aforementioned light impeding events is higher for 30 kGy samples as compared to the 100 kGy one because of radiation induced free radical oxidation degradation [21,34].

The effective penetration depth (δ in mm) is a measure of how deep light or any electromagnetic radiation can penetrate. It is defined as the depth at which the intensity of the radiation inside the material falls to 1/e (about 37%) of its original value at (or more properly, just beneath) the surface. The penetration depth is calculated as follows:

δ = 1/µeff(4)

where:

(5) $$\mu_{eff}=\sqrt{\mu_{a}+\mu_{s}{\left(1-g\right)}_{}}$$

**Figure 4:**
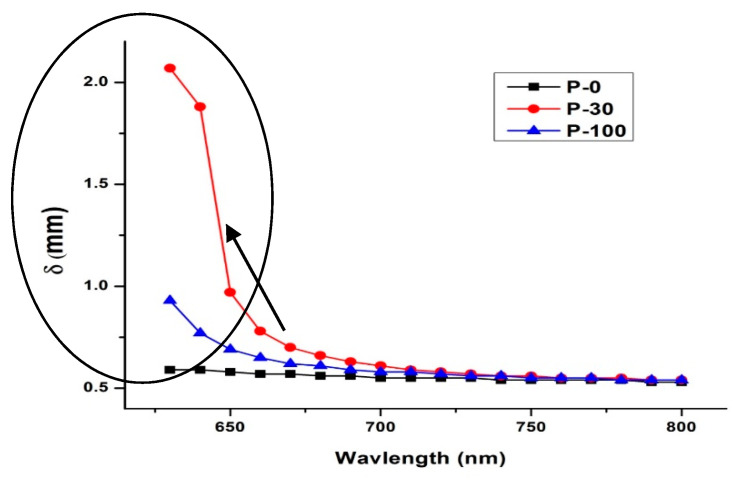
Penetration depth for the sample UHMWPE over the wavelength of interest from 600 nm to 800 nm.

Figure 4 represents the variation of δ as a function of incident wavelength and absorbed dose. The values for pristine UHMWPE are constant over the wavelength of interest; however, increases up to approximately 0.9 mm for the 100 kGy irradiated sample and up to 2 mm for the 30 kGy sample. As the attenuation coefficient ‘μ_t_’ (sum of absorption and scattering coefficients) is the major responsible factor in reducing the intensity of the incident beam. Therefore, the increase in the values of penetration depth δ for irradiated samples (more specifically for the 30 kGy sample) (see Figure 4) is quite justified and understandable.

### 3.4. Theoretical Validation of Extracted Optical Properties

Theoretical validation of the extracted optical properties is performed by using the output of the KMM model as an input of the MCML model, which is the general-purpose Monte Carlo-based code to simulate the light propagation through turbid media. The Monte Carlo-based simulation program, MCML, for the infinitely narrow beam is available at the website https://omlc.org/software/mc/ (assessed on 23 November 2019) [45]. This code is used in this study to simulate the light by getting experimental results from the KMM model. The code simulates the light transport in the xyz-planes while considering the sample surfaces the x- and y-axes, and depth z-axis [46,47].

**Figure 5:**
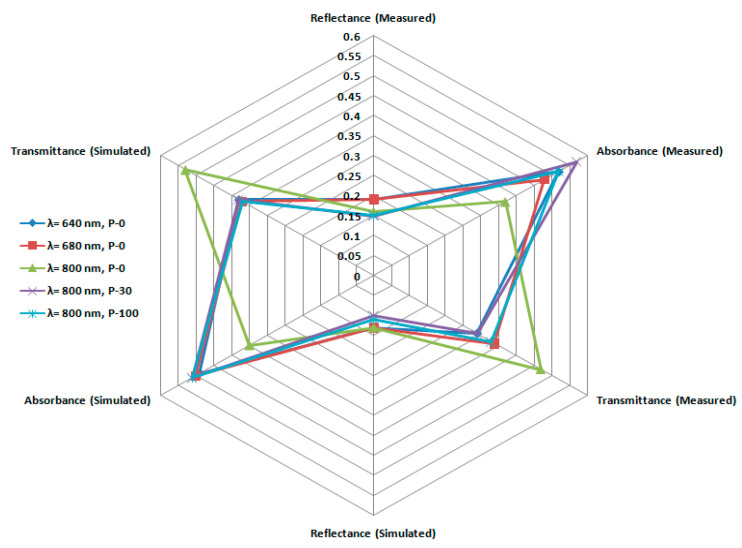
Radar Plot for representing the theoretical and experimental data validation.

In order to simulate a normal incident photon beam, the cosines direction are first defined as r_x_ = 0, r_y_ = 0, r_z_ = 1 along x–y emission plane with polar angle θ directed normal to the surface. To define each photon emission, an emission cone having azimuthal angle φ is used. The four-dimensional functions that are recorded in the course of this study are absorption, transmittance, diffusion, and reflectance. The sample thickness that is used as an input is 0.05 cm, i.e., similar to that taken in the experimental measurements. The direction and emission position are split into 200 bins of 50 × 10^−4^ cm each on the x–y plane, having 30 bins regarding θ ∈ [0, π/2] and 48 bins for ϕ ∈ [0, 2π]. For each run, 5 × 10^8^ photons (each with unit weight) are used for the calculation of absorption, transmittance, and reflectance. For validation, the theoretical results are compared with the experimental ones and are shown in Figure 5 as a radar plot.

The simulated physical properties (transmittance, reflectance, and absorbance) at 800 nm for pristine, 30 kGy, and 100 kGy irradiated samples are then plotted against the measured ones on a radar plot as shown in Figure 5. The symmetry of the trend about the central axis of the plot is evident (see Figure 5). This confirms the excellent correspondence between experimental and simulated results with a relative difference of less than 5% among the experimental and theoretical values.

### 3.5. Effect of Radiation on Light Distribution

**Figure 6:**
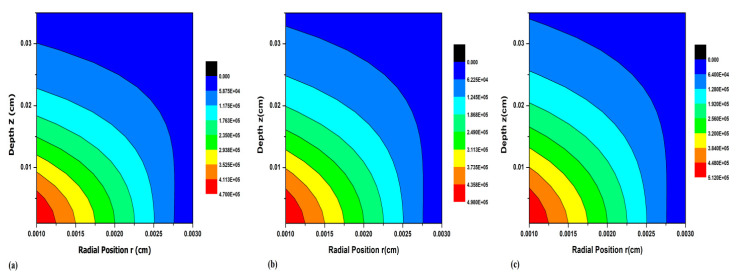
Photon distribution representation as a function of depth (z) and radial position (r) at 800 nm for (**a**) pristine UHMWPE (**b**) 30 kGy irradiated UHMWPE, and (**c**) 100 kGy irradiated UHMWPE.

After being confident that the extracted optical parameters are correct and that the theoretical results of reflectance, transmittance, and absorbance generated (with these extracted optical properties as input) with Monte Carlo model for light transport from turbid media are in good agreement with the measured values, the photon distribution within the UHMWPE is modeled. The results of photon absorption as a function of z and r at 800 nm for pristine, 30 kGy irradiated, 100 kGy irradiated UHMWPE are shown in Figure 6. The noteworthy trends in the above figure are the increase in penetration depth and photon absorption per unit area with irradiation. To have a clearer insight, one can see the end of the light blue colored contour, which ends at 0.03 cm for pristine UHMWPE samples at 0.32 cm for 30 kGy irradiated samples, and at 0.034 cm for 100 kGy irradiated samples. Furthermore, the absorbed number of photons in the first contour for P-0, P-30, and P-100 are 4.70 × 10^5^ cm^−2^, 4.98 × 10^5^ cm^−2^, and 5.12 × 10^5^ cm^−2^, respectively. The effect of radiation on the transport albedo, which is the ratio of scattering coefficients to the attenuation coefficients, can be used to explain the photon distribution characteristics of UHMWPE. The values of transport albedo vary from 0 to 1. The transport albedo = 0 means that no light will pass through the sample while transport albedo = 1 means the incident light passes through the sample without any absorption. It can be seen from Table 1 that transport albedo is 0.9545 for P-0 and its value reduces to 0.9392 with irradiation, which is evidence of the fact that photon distribution per unit area, i.e., number of photon/cm^2^ is higher for irradiated samples. The photon distribution representations shown in Figure 6, i.e., the plots of absorption as a function of wavelength and radiation, are in favor of our argument that transport albedo is an important factor to consider when using light as an alternative for UHMWPE modification and/or stabilization.

## 4. Conclusions

In this study, the effects of gamma irradiation and absorbed dose on the optical properties (absorption and scattering coefficients) of UHMWPE from 600 nm to 800 nm are explored. The optical characteristics of pristine and gamma irradiated UHMWPE sample were extracted using the KMM model in conjugation with measured values of diffuse reflectance and transmittance. The results reveal a significant decrease in coefficient (μ_a_ & μ_s_) values for irradiated samples at lower wavelengths, i.e., from 630 nm to 700 nm. This decrease in coefficients for the 30 kGy irradiated sample is higher as compared to the 100 kGy irradiated one and is attributed to the higher oxidation damage of UHMWPE at this dose value. The slight increase in the values of scattering coefficients μa on moving from 700 nm to 800 nm for irradiated samples is attributed to dominant scattering and small absorption of polyethylene on near IR and IR absorption. Furthermore, light distribution at 800 nm is modelled for pristine, 30 kGy and 100 kGy gamma-irradiated samples using Monte Carlo methods. The extracted scattering and absorption coefficients are used as inputs for simulating the 5 × 108 photons (each with unit weight) for each run. The results reveal an increase in the penetration depth and photon absorption per unit area with irradiation, which is attributed to a decrease in transport albedo, i.e., the ratio of scattering coefficients to the attenuation coefficients with irradiation. We thus conclude that transport albedo is the prime and most important factor to be considered before using a suitable light wavelength as an alternative for the modification and/or stabilization of UHMWPE.

## Figures and Tables

**Table 1:** Optical properties of UHMWPE samples at particular wavelengths.

λ (nm)	Absorption Coefficient μ_a_ (cm^−1^)			Scattering Coefficient μ_s_ (cm^−1^)			Attenuation Coefficient μ_t_ (cm^−1^)		
	P-0	P-30	P-100	P-0	P-30	P-100	P-0	P-30	P-100
630	5.34	1.67	3.69	120.06	29.72	66.35	125.39	31.39	70.04
640	5.44	1.84	4.48	121.17	32.28	79.63	126.61	34.12	84.11
650	5.53	3.59	4.98	122.29	61.83	87.58	127.82	65.43	92.57
660	5.62	4.50	5.30	122.92	76.30	92.61	128.54	80.81	97.92
670	5.68	5.01	5.54	123.54	83.97	96.01	129.22	88.99	101.55
680	5.74	5.32	5.73	124.04	88.71	98.45	129.79	94.03	104.18
690	5.77	5.60	5.86	124.78	92.26	100.30	130.55	97.86	106.16
700	5.80	5.80	5.95	125.38	94.93	102.11	131.19	100.74	108.07
710	5.87	5.97	6.03	125.42	96.73	103.41	131.29	102.70	109.45
720	5.88	6.07	6.11	126.03	98.11	104.23	131.91	104.18	110.34
730	5.91	6.18	6.19	126.84	99.92	105.72	132.75	106.11	111.92
740	5.95	6.29	6.25	126.85	100.57	106.35	132.81	106.86	112.60
750	5.99	6.37	6.32	127.01	101.36	106.89	133.00	107.73	113.21
760	6.04	6.46	6.38	126.73	101.62	107.24	132.77	108.09	113.63
770	6.04	6.49	6.41	127.27	102.19	107.65	133.32	108.68	114.06
780	6.06	6.55	6.44	127.60	102.48	108.08	133.66	109.038	114.53
790	6.07	6.59	6.46	127.86	102.71	108.47	133.94	109.31	114.94
800	6.09	6.65	6.47	128.02	102.78	109.11	134.12	109.43	115.59

## Data Availability

Data will be provided on demand.
